# Supplementary material for: Evaluation of albumin use in a community hospital setting: A retrospective study looking at appropriate use and prescribing patterns
Source: PLoS One. 2021 Oct 6;16(10):e0257858. doi: 10.1371/journal.pone.0257858 (PMC8494356; doi:10.1371/journal.pone.0257858)
Supplement: Attachment — Submitted filename: Response to Reviewers.docx [file pone.0257858.s002.docx]

August 31, 2021

To the manuscript reviewers:

Thank you for taking the time to review our manuscript and I appreciate your time and feedback. I would like to take this opportunity to review and provide the answers I can to the points that were brought up during the review process of this manuscript.

**Reviewer 1:**

- **Samuel and Coyle described the use of human albumin solution (HAS) in a community hospital. Overall, the manuscript is a clearly written however I am uncertain as to why the results warrant a publication. In its present form, the work resembles an audit rather than original research.**

This research was completed as part of a medication use evaluation and quality improvement project. The goal of this evaluation was to determine from which departments most of the albumin orders originate for a community hospital and to determine for what indications is it currently used. The impact of the article is to reflect albumin use in a community hospital and to act as a pilot study to determine if general guidelines of albumin use should be updated.

- **The introduction does not paint a convincing picture as to why the research question is important. Furthermore, why should prescribing practices of HAS vary between tertiary hospitals and community hospitals if FDA approval for HAS is standard; the authors do not discuss how their results differ from that of current literature. As such, I struggle to see where the novelty lies.**

I believe these results found in this research warrant a publication because there is less data showing how albumin is used overall in a system. Multiple guidelines for albumin use have been published and utilized within multiple hospital settings over the years, but we still see albumin used more often and outside of the parameters set forth in these guidelines. I agree with the reviewer that the prescribing practices do not differ between a community hospital setting versus a tertiary hospital setting, however, this research may point to the fact that there is a potential discrepancy between what is in the guidelines and clinical practice. This manuscript looks at the distribution of albumin within the current community healthcare setting, without preference for one clinical area. It can also serve as a starting point for future research into which departments’ albumin use should be more scrutinized.

- **To be considered for publication, the manuscript is in need of a Major revision. The introduction and discussion needs to be improved and more evidence provided. 9 references is insufficient for an original research article.**

I have added more content to the introduction and discussion sections of the attached manuscript.

- **"The results could be used to influence prescribing practices in the health care system and decrease cost to the hospital and the patient."I am unsure how describing the use of HAS in a community hospital can influence "prescribing practices in the health care system and decrease cost to the hospital and patient". It is not clearly explained how this can be achieved with the results herein.**

The research works best as a cross-sectional review of how albumin is typically used within a given healthcare system. From these results, other healthcare systems may be able to decide to pursue research into certain departments regarding the necessity of albumin use.

- **There is mention of standard deviations although I do not reported in the results.**

Standard deviations have been added to applicable results.

- **Observational studies should be reported using the STROBE checklist. There are missing segments in the methods e.g. inclusion and exclusion criteria.**

The changes to the methods section were completed and added to the manuscript according to the STROBE checklist, as applicable.

- **Why wasn't data on the adverse reactions of HAS investigate? Were there any differences in the approved and "off label" use?**

The safety data was not reviewed due to the scope of the research project. It is unknown at this time if there was any difference in safety data between approved and “off-label” use.

- **I do not see how these results are different from data that is already available in literature.**

These results mainly show the distribution of albumin use in the healthcare setting, revealing that regardless of hospital size, albumin is still widely used, despite crystalloid availability. The project was designed as a reflection of how albumin is prescribed in the modern hospital setting, even with the available guidelines and literature.

- **The discussion is very superficial and not impactful.**

I have updated the discussion with more evidence in the attached, updated manuscript.

**Reviewer 2:**

- **Such kind of analyses have been published before for other health care settings and larger patient populations. A relatively small sample size is the major limitation and prevents novel insight or meaningful conclusions.**

I agree that one of the major limitations with this article is the small sample size which prevents novel insight. Our research may be used as a preliminary look at one community hospital setting on which other studies may be conducted to evaluate albumin use in a larger healthcare setting with a wider patient population.

- **Questions regarding use of albumin arise particularly in controversial issues such as combinations of a particular clinical condition with significant hypoalbuminemia with and without hypovolemia or when 5% vs. 20% to 25% human albumin solutions are used. Inappropriate use in non-cardiac surgery would merit further analysis, however, sample size is too small.**

This research was mainly used to evaluate prescribing patterns to determine from where most of the orders for albumin were originating and to see if the guidelines are followed within the healthcare setting. Some of the subpopulations within this dataset could be used, and future research could expand on the findings brought up in this paper. This should be considered as a pilot study, used to to provide an estimate of albumin use within a community hospital over a relatively short period of time as a quality improvement project.

Thank you again for your feedback and your time in reviewing this. Please find attached a revised manuscript with the recommended changes. Please do not hesitate to reach out if there is anything further that I can clarify. Thank you.

Sincerely,

Timothy Coyle, PharmD
